# *In vitro *suppression of the *MMP-3 *gene in normal and cytokine-treated human chondrosarcoma using small interfering RNA

**DOI:** 10.1186/1749-799X-4-45

**Published:** 2009-12-24

**Authors:** Korakot Nganvongpanit, Patama Chaochird, Puntita Siengdee, Peraphan Pothacharoen, Kasisin Klunklin, Siriwadee Chomdej, Supamit Mekchay, Prachya Kongtaweelert

**Affiliations:** 1 Bone and Joint Research Laboratory, Department of Veterinary Biosciences and Public Health, Faculty of Veterinary Medicine, Chiang Mai University, Chiang Mai 50100, Thailand; 2 Department of Biology, Faculty of Science, Chiang Mai University, Chiang Mai 50200, Thailand; 3 Thailand Excellence Centre for Tissue Engineering, Department of Biochemistry, Faculty of Medicine, Chiang Mai University, Chiang Mai 50200, Thailand; 4 Department of Orthopedics, Faculty of Medicine, Chiang Mai University, Chiang Mai 50200, Thailand; 5 Department of Animal Science, Faculty of Agriculture, Chiang Mai University, Chiang Mai 50200, Thailand

## Abstract

**Background:**

Matrix metalloproteinase (MMPs) synthesized and secreted from connective tissue cells have been thought to participate in degradation of the extracellular matrix. Increased MMPs activities that degrade proteoglycans have been measured in osteoarthritis cartilage. This study aims to suppress the expression of the *MMP-3 *gene in *in vitro *human chondrosarcoma using siRNA.

**Methods:**

Cells were categorized into four groups: control (G.1); transfection solution treated (G.2); negative control siRNA treated (G.3); and *MMP-3 *siRNA treated (G.4). All four groups were further subdivided into two groups - treated and non-treated with IL-1β- following culture for 48 and 72 h. We observed the effects of gene suppression according to cell morphology, glycosaminoglycan (GAG) and hyaluronan (HA) production, and gene expression by using real-time polymerase chain reaction (PCR).

**Results:**

In IL-1β treated cells the apoptosis rate in G.4 was found to be lower than in all other groups, while viability and mitotic rate were higher than in all other groups (*p *< 0.05). The production of GAG and HA in G.4 was significantly higher than the control group (*p *< 0.05). *MMP-3 *gene expression was downregulated significantly (*p *< 0.05).

**Conclusion:**

*MMP-3 *specific siRNA can inhibit the expression of *MMP-3 *in chondrosarcoma. This suggests that *MMP-3 *siRNA has the potential to be a useful preventive and therapeutic agent for osteoarthritis.

## Background

Osteoarthritis (OA) is the most common disease in joints. The incidence of OA in humans is 12.1% of the population between 25-74 years of age [1]. Reports on OA epidemiology consistently show an almost exponential increase of prevalence with increasing age [2]. OA is not restricted to humans only; it is also an important problem in veterinary medicine, particularly for racehorses and dogs [3].

Nowadays, gene therapy offers novel approaches to the medical management of OA [3,4]. One of the latest techniques is RNA interference (RNAi), which is widely used to downregulate the mRNA level of a particular gene. RNAi is the process of sequence-specific, post-transcriptional gene silencing in animals and plants, initiated by double-stranded RNA (dsRNA) that is homologous in sequence to the silenced gene [5,6]. The mediators of sequence-specific messenger RNA degradation are 21- and 22-nucleotide small interfering RNAs (siRNAs) generated by ribonuclease III cleavage from longer dsRNAs [7-9].

In this research, we suppressed the *matrix metalloproteinase-3 *(*MMP-3*) gene. The enzymes in the matrix metalloproteinase group (MMPs) play an important role in articular cartilage degradation [10,11]. MMP-3 acts to degrade the extracellular matrix (ECM): proteoglycans, gelatin, laminin, fibronectin and collagen (types III, IV and IX) [12]. In addition, MMP-3 can stimulate the other enzymes in the MMPs group, such as MMP-1, MMP-7, MMP-8, MMP-9 and MMP-13 [13]. This stimulation increases biochemical substance degradation, including degradation of type II collagen, the most important type of collagen in the ECM. This research also focuses on the effect of the suppression of the *MMP-3 *gene on mRNA and proteoglycan production. Moreover, the biological effects of the suppression of this gene in chondrosarcoma cells will be assessed during cell culture *in vitro*.

## Methods

### Experimental design

In the experiment, cells were divided into four groups: group 1 (G.1) was a control group; group 2 (G.2) added only a transfection solution; group 3 (G.3) added a negative control siRNA; and group 4 (G.4) was an experimental group with added *MMP-3 *specific siRNA. All four groups were then divided into subgroups, non-treated and treated (for 24 h) with 10 ng/ml recombinant human IL-1β (R&D Systems; Minneapolis MN, USA).

Assessment of the results was performed at 48 and 72 h following treatment. Observations included cell morphology (viability, and rates of mitotis and apoptosis), hyaluronan (HA) and glycosaminoglycan (GAG) synthesis, and gene expression.

### Cell culture

Samples of the human chondrosarcoma cell line (sw1353) were obtained from the Thailand Excellence Center for Tissue Engineering, Department of Biochemistry, Faculty of Medicine, Chiang Mai University, Chiang Mai, Thailand. The cells were maintained in Dulbecco's modified Eagle's medium (DMEM; GIBCO^® ^Invitrogen; Carlsbad CA, USA) supplemented with 10% fetal calf serum (FCS), 100 units/ml penicillin and 100 μg/ml streptomycin (GIBCO^® ^Invitrogen), and then cultivated in a CO_2 _incubator (5% CO_2_, 37°C).

When the cells had reached confluence, the media was removed and the cells washed in 10 ml Hanks' balanced salt solution (HBSS; BioWhittaker™ Cambrex Bio Science; Verviers, Belgium) to remove traces of FCS. After removing HBSS, the cells were trypsinized with 3 ml of trypsin-EDTA (BioWhittaker™ Cambrex Bio Science). After examining the cells using an inverted microscope to ensure that all cells were detached and floating, 7 ml of fresh complete media was added. The media plus trypsinized cells were divided among an appropriate number of flasks (depending on the desired splitting ratio) and the volume in each flask was raised up to 10 ml with the addition of fresh complete media.

Cells for pellet culture were trypsinized, and the total number of cells was calculated based on a hemocytometer count: 1 × 10^6 ^cells/pellet, cultured in 1 ml pellet media (DMEM supplemented with 10% FBS and pen/strep which contained 10^7 ^M dexamethasone, 25 μg/ml L-ascorbate 2-phosphate and 1× ITS 1+). The growth factor pellet media was basic pellet media plus 100 ng/ml IGF-1 and 10 ng/ml TGFβ3 [14].

### siRNA template design and siRNA transfection

*MMP-3 *synthetic siRNA were designed by Qiagen (Qiagen; Hilden, Germany) using the BIOPRED algorithm licensed from Novartis. A BLAST (Basic Local Assignment Search Tool) search was conducted on the sequence to ensure gene specificity. Template oligonucleotides were synthesized by HP GenomeWide siRNA (Qiagen). The negative non-silencing control siRNA (Qiagen) has no homology to any known mammalian gene, and is used to control for nonspecific silencing effects. If altered expression or phenotypes are observed in cells transfected with negative control siRNA, these changes will be nonspecific. Transfection of siRNA was carried out using the HiPerFect Transfection Reagent (Qiagen). Briefly, 1 × 10^6 ^cells per 100 mm dish were seeded in 7000 μl of DMEM, and incubated under normal growth conditions (typically 37°C and 5% CO_2_). Then a transfection complex was prepared by diluting 600 ng siRNA in 1000 μl culture medium without serum, and then adding 40 μl of HiPerFect transfection reagent to the diluted siRNA. This was then mixed by vortexing. The complexes were added drop-wise onto the cells, and the plates were then gently swirled to ensure uniform distribution of the transfection complexes. Cells were incubated with the transfection complexes under normal growth conditions, and gene silencing was monitored 48 and 72 h after transfection. Transfection efficiency was evaluated with fluorescein siRNA using fluorescence microscopy at 24 h after transfection.

### Determination of cell viability and cell apoptosis

Cell viability was determined by the Trypan blue dye exclusion method. After 5 min of incubation with 0.4% Trypan blue, the percentages of stained cells (indicative of nonviable cells) versus stain-excluding cells were counted in a hemocytometer. Then the percentage of viable cells was calculated as follows: viable cells (%) = (total number of viable cells × 100)/total number of cells.

For measurement of cell apoptosis, cells were seeded into 8-well chamber slides (300 μl cell suspension/well). When confluent, cell survival was assessed by staining cell nuclei with the vital DNA-binding dye Hoechst 33342 (Sigma; Thailand). The slides were incubated at 37°C for 30 min. Cultures were then washed three times with phosphate-buffered saline, and examined by an inverted fluorescence microscope. Dead cells were readily recognized, as they had a condensed or fragmented nucleus. Then the percentage of apoptosis cells was calculated as follows: apoptosis cell (%) = (total number of apoptosis cells × 100)/total number of cells.

Morphology of the cells was studied by using aceto-orcein dyes that can separate mitotic cells from interphase cells. Then the percentage of mitotic cells was calculated as follows: mitotic index (%) = (total number of mitotic cells × 100)/total number of cells.

### Measurement of proteoglycan levels

Proteoglycans in this study were GAG and HA. These markers can indicate the alteration of the biochemical composition of chondrocytes, including chondrosarcoma cells which were used in this study [14,15]. The cells were cultured for 48 and 72 h before being collected and stored in culture media at -20°C.

### Measurement of GAG levels

The level of GAG appearing in the medium of explants, cell cultures, and papain-digested cells was determined using the dimethylmethylene blue (DMMB) assay for sulfated glycosaminoglycan 10 using chondroitin sulfate C (shark cartilage extract; Sigma-Aldrich, USA) as standard. The DMMB solution was added to the diluted sample and standard and appropriate blank solution prior to absorbance reading at 525 nm in a microplate reader spectrophotometer.

### Measurement of HA levels

HA in medium and cell layer was measured using a competitive inhibition-based ELISA as previously described, with modifications [14,15]. Briefly, culture media or papain-digested samples (175 μl) containing unknown amounts of HA, as well as a standard containing known concentrations of a highly purified HA preparation (Healon^®^, Pharmacia AB; Uppsala, Sweden), were placed in small polypropylene tubes with appropriate concentrations of biotinylated-HA binding proteins (B-HABP) (175 μl) and incubated at room temperature (25°C) for 1 h. Aliquots (100 μl) of this reaction mixture were applied to microplates coated with human umbilical cord HA (and BSA-blocked), and incubated at 25°C for 1 h. The wells were then washed with phosphate-buffered saline solution containing 0.05% Tween-20. The appropriate dilution (1:2000 in PBS) of anti-biotin peroxidase conjugate (Zymed Laboratories, Inc.; San Francisco CA, USA) was then added to each well, incubated at 25°C for 1 h, and washed, after which peroxidase substrate (OPD, o-phenylenediamine) was added. After incubation at 25°C for 20 min, the reaction was stopped by the addition of 50 μl 4 M H_2_SO_4_. The absorbance ratio at 492/690 nm was measured using a Titertek Multiskan M340 microplate reader. HA concentration in the culture media samples was calculated relative to a standard curve generated from the purified HA preparation.

### RNA isolation, synthesis of cDNA

RNA isolation and purification in each group was performed using an RNeasy Mini Kit protocol (Qiagen), including the DNA removal step, according to the manufacturer's guidelines. RNA was eluted in 40 μl of RNase-free water (Qiagen).

Reverse transcription was performed using 10 μl RNA with oligo(dT)12-18 and Superscript II reverse transcriptase (Invitrogen; Karlsruhe, Germany). In terms of the order of adding reaction components, mRNA and oligo(dT) primer were mixed first, heated to 70°C for 3 min, and placed on ice until the addition of the remaining reaction components. The reaction was incubated at 42°C for 90 min, and terminated by heat inactivation at 70°C for 15 min.

### Quantitative real-time PCR

Quantification of *MMP-3 *and *glyceraldehyde-3-phosphate dehydrogenase *(*GAPDH*) mRNA in the cells of each treatment group was assessed by real-time quantitative PCR. Moreover, five transcripts related to the cell - including *tissue inhibitor of metallopeptidase-3 *(*TIMP-3*); *hyaluronan synthase 1 *(*HAS-1*); *HAS-2*; *aggrecan *(*AGG*); and *collagen, type 2, alpha 1 chain *(*COL2A1*) - were also quantified in all four groups to check the specificity of mRNA suppression by the siRNA. An ABI Prism^® ^7000 apparatus (Applied Biosystems; Foster City CA, USA) was used to perform the quantitative analysis using SYBR^® ^Green JumpStart™ Taq ReadyMix™ (Sigma) incorporation for dsDNA-specific fluorescent detection dye. Quantitative analyses of cell *MMP-3*, *TIMP-3*, *HAS-1*, *HAS-2*, *AGG *and *COL2A1 *cDNA were performed in comparison with *GAPDH *as an endogenous control [16,17], and were run in separate wells. PCR was performed by using 2 μl of each sample of cDNA and specific amplification primers. The primer sequences were designed for PCR amplification according to the human cDNA sequence (Table 1) using Primer Express^® ^Software v2.0 (Applied Biosystems). Standard curves were generated for both target and endogenous control genes using serial dilution of plasmid DNA (10^1 ^- 10^8 ^molecules). The PCRs were performed in 20 μl reaction volume containing 10.2 μl SYBR^® ^Green universal master mix (Sigma), optimal levels of forward and reverse primers, and 2 μl of embryonic cDNA. During each PCR, reaction samples from the same cDNA source were run in duplicate to control the reproducibility of the results. A universal thermal cycling parameter (an initial denaturation step at 95°C for 10 min, and 45 cycles of denaturation at 95°C for 15 s and 60°C for 60 s) was used to quantify each gene of interest. After the end of the last cycle, a dissociation curve was generated by starting the fluorescence acquisition at 60°C and taking measurements at 7 sec intervals until the temperature reached 95°C. Final quantitative analysis was done using the relative standard curve method, as used in Nganvongpanit et al. (2006) [16,17]. Results are reported as the relative expression level compared to the calibrator cDNA after normalization of the transcript amount to the endogenous control.

**Table 1:** Set of primers used for real-time quantitative PCR

Gene	Primer sequences	Ta* (°**C)**	Amplicon size (bp)
*MMP-3 *(NM_002422)	Forward: 5'-CTTTTGGCGAAAATCTCTCAG-3' Reverse: 5'-AAAGAAACCCAAATGCTTCAA-3'	55	404
*TIMP-3* (NM_000362)	Forward: 5'-AACTCCGACATCGTGATCCG-3' Reverse: 5'-GTAGTAGCAGGACTTG ATCT-3'	59	347
*COL2A1* (NM_001844)	Forward: 5'-CAACACTGCCAACGTCCAGAT-3' Reverse: 5'-CTGCTTCGTCCAGATAGGCAAT-3'	59	106
*AGG* (NM_013227)	Forward: 5'-ACTTCCGCTGGTCAGATGGA-3' Reverse: 5'-TCTCGTGCCAGATCATCACC-3'	59	110
*HAS-1* (NM_001523)	Forward 5'-CGGCCTGTTCCCCTTCTTCGTG-3' Reward 5'-TCGTGTGCTACGCTGCGGACCA-3'	65	348
*HAS-2* (NM_005328)	Forward 5'-CACAGCTGCTTATATTGTTG-3' Reward 5'-AGTGGCTGATTTGTCTCTGC-3'	51	358
*GAPDH* (NM_002046)	Forward: 5'-TGGTATCGTGGAAGGACTCAT-3' Reverse: 5'-GTGGGTGTCGCTGTTGAAGTC-3'	58	370

* Ta = Annealing temperature

### Statistical analysis

Results of cells morphology and proteoglycans were displayed as mean ± SD. The mRNA expression analysis for studied genes in all treatment groups was based on the relative standard curve method. All data were analyzed using the Statistical Analysis System (SAS) version 8.0 (SAS Institute, Inc.; Cary NC, USA) software package. Differences in mean values between two or more experimental groups or developmental stages were tested using ANOVA followed by multiple pairwise comparisons using a t-test. Differences of *p *< 0.05 were considered to be significant.

## Results

### Effect of IL-1β treatment

Treatment of cells using 10 ng/ml IL-1β for 24 h had an effect (*p *< 0.05) on cell morphology, proteoglycan production and gene expression (Table 2).

**Table 2:** Effect of IL-1β treatment in *in vitro *chondrosarcoma culture

	48 h		72 h	
	
	Non-IL1	IL-1	Non-IL1	IL-1
**Cell morphology**				
Viability rate	93.05 ± 4.23	46.41 ± 6.04*	93.87 ± 3.44	26.52 ± 5.97*
Apoptosis rate	2.28 ± 1.71	63.39 ± 11.43*	4.62 ± 1.72	70.73 ± 5.02*
Mitotic rate	9.42 ± 2.43	1.36 ± 0.90*	16.22 ± 7.62	0.85 ± 0.30*
**Proteoglycan production**				
HA (ng/ml)	121.04 ± 23.03	48.54 ± 12.85*	146.04 ± 11.93	35.29 ± 8.54*
GAG (μg/ml)	1.91 ± 0.88	0.56 ± 0.14*	1.72 ± 0.81	0.17 ± 0.07*
**Relative gene expression**				
*MMP-3*	0.98 ± 0.07	2.03 ± 0.23*	1.56 ± 0.18	2.94 ± .019*
*TIMP-3*	1.01 ± 0.03	0.57 ± 0.21*	1.35 ± 0.09	0.34 ± 0.06*
*HAS-1*	1.02 ± 0.13	0.37 ± 0.15*	1.35 ± 0.10	0.15 ± 0.07*
*HAS-2*	1.02 ± 0.11	0.10 ± 0.04*	1.18 ± 0.12	0.05 ± 0.04*
*AGG*	1.01 ± 0.06	0.43 ± 0.18*	1.35 ± 0.10	0.17 ± 0.03*
*COL2A1*	1.02 ± 0.11	0.15 ± 0.10*	1.48 ± 0.14	0.03 ± 0.08*

A significant difference (*p *< 0.05) between treatment and non-treatment with IL-1β at the same period (48 or 72 h) is displayed with superscript (*) on the number.

Viability and mitotic rates in IL-1β treated groups were decreased compared to the non-treated groups (*p *< 0.05). But the apoptosis rate in IL-1β treated groups was increased (*p *< 0.05). The level of proteoglycan production (HA and GAG) decreased compared to groups not treated with IL-1β (*p *< 0.05). The relative expression of *MMP-3 *in IL-1β treated groups was found to be increased (*p *< 0.05) compared to the non-treated groups, while the other genes (*TIMP-3*, *HAS-2*, *HAS-2*, *AGG *and *COL2A1*) were decreased (*p *< 0.05).

### Effect of MMP-3 siRNA on cell morphology

#### Viability rate

**Figure 1:**
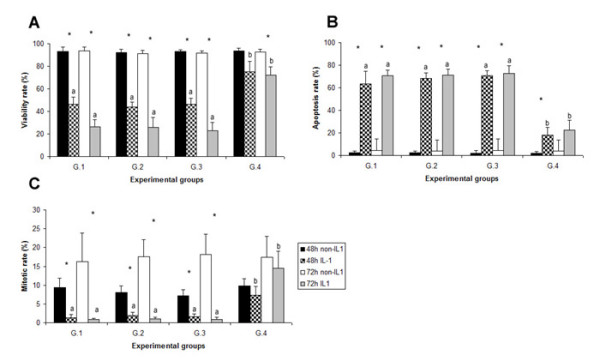
**Viability (A), apoptosis (B) and mitotic (C) rate in all experimental groups**. Individual bars show the mean ± SD. A significant difference (*p *< 0.05) between the four groups at the same condition (48 h non-IL1, 48 h IL1, 72 h non-IL1 and 72 h IL1) is displayed with superscript (^a, b^) on the bars. A significant difference (*p *< 0.05) between treatment and non-treatment with IL-1β at the same period (48 or 72 h) is displayed with superscript (*) on the bars. G1 = control group; G.2 = solution control; G.3 = non-silencing siRNA; and G.4 = *MMP-3 *siRNA.

In order to examine cell viability, chondrosarcoma cells were stained with Trypan blue for 5 min and then examined under a light microscope. No significant differences were detected in the four groups of chondrosarcoma which were not treated with IL-1β (Fig. 1). These results indicate that IL-1β treatment in all groups significantly decreased cell viability (*p *< 0.05). *MMP-3 *siRNA treatment resulted in significantly increased (*p *< 0.05) cell viability in IL-1β treated groups, both at 48 and 72 h.

#### Apoptosis rate

The effect of siRNA on cell apoptosis was detected by staining the nuclei with Hoechst 33342 and then examining the cells under a fluorescence microscope. Cells treated with IL-1β had a significantly increased apoptosis rate (*p *< 0.05). In cells not treated with IL-1β, no significant difference was observed (*p *> 0.05) between any of the groups. In this experiment, the apoptosis rate in G.4 at both 48 and 78 h was significantly lower than in the other groups (Fig. 1).

### Mitotic rate

Cells treated with IL-1β had a significantly decreased mitotic rate (*p *< 0.05). In cells not treated with IL-1β, no significant difference was observed (*p *> 0.05) between groups. In this experiment, the apoptosis rates in G.1, G.2 and G.3 of the IL-1β treated groups (at both 48 and 78 h) were significantly lower than the non-treated groups (Fig. 1). But this difference was not found in G.4.

### Proteoglycan levels

**Figure 2:**
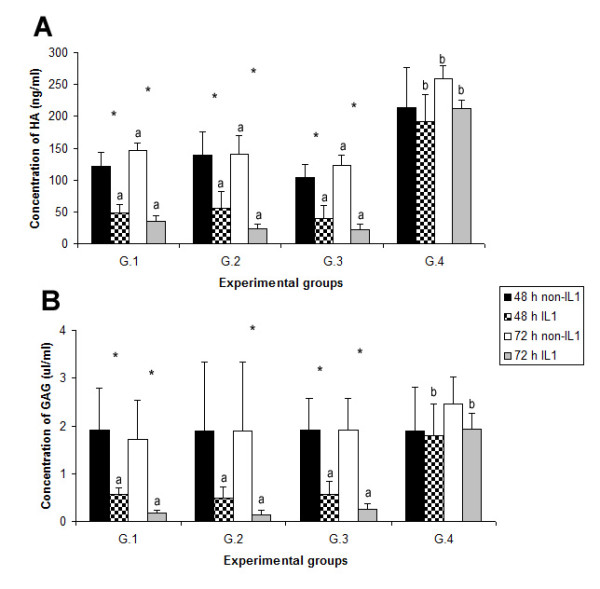
**Levels of hyaluronan (HA) and glycosaminoglycan (GAG) in all experimental groups**. Individual bars show the mean ± SD. A significant difference (*p *< 0.05) between the four groups at the same condition (48 h non-IL1, 48 h IL1, 72 h non-IL1 and 72 h IL1) is displayed with superscript (^a, b^) on the bars. A significant difference (*p *< 0.05) between treatment and non-treatment with IL-1β at the same period (48 or 72 h) is displayed with superscript (*) on the bars. G1 = control group; G.2 = solution control; G.3 = non-silencing siRNA; and G.4 = *MMP-3 *siRNA.

The levels of HA and GAG are shown in Fig. 2. In the 72 h culture non-treated with IL-1β, the level of HA in G.4 was found to be increased 170% (*p *< 0.05) compared to G.1. After treatment of cells with IL-1β, the level of GAG in G.4 compared to G.1 was significantly increased: 120% and 143% in 48 h and 72 h cultures, respectively. The level of HA also increased 400% and 600% in 48 h and 72 h cultures, respectively.

### The effect of siRNA on target mRNA expression

**Figure 3:**
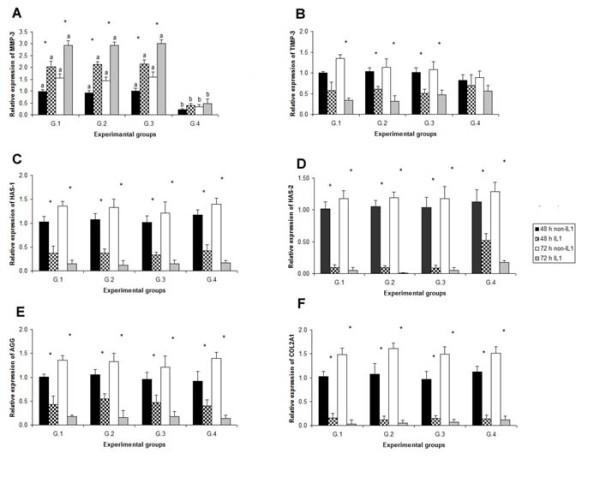
**Relative expression of *MMP-3 *(A), *TIMP-3 *(B), *HAS-1 *(C), *HAS-2 *(D), *AGG *(E) and *COL2A1 *(F) in all experimental groups**. Individual bars show the mean ± SD. A significant differences (*p *< 0.05) between the four groups at the same condition (48 h non-IL1, 48 h IL1, 72 h non-IL1 and 72 h IL1) is displayed with superscript (^a, b^) on the bars. A significant difference (*p *< 0.05) between treatment and non-treatment with IL-1β at the same period (48 or 72 h) is displayed with superscript (*) on the bars. G.1 = control group; G.2 = solution control; G.3 = non-silencing siRNA; and G.4 = *MMP-3 *siRNA.

The specificity of *MMP-3 *siRNA was determined by transfection of non-targeted siRNA as a control. Moreover, the selective suppression efficiency of *MMP-3 *siRNA was assessed by analyzing the expression levels of other independent but functionally related transcripts (*TIMP-3*, *HAS-1*, *HAS-2*, *AGG *and *COL2A1*) in the same stages of all four groups. The results of this mRNA quantification show that *MMP-3 *siRNA triggered a remarkable suppression in the amount of *MMP-3 *mRNA in the cells. As shown in Fig. 3, the relative expression level of *MMP-3 *mRNA in G.4 at 48 and 72 h was found to be reduced by 80% compared to G.1 (*p *< 0.05).

With the aim of investigating the specificity of *MMP-3 *siRNA in the suppression of the target mRNA, five functionally related transcripts were analyzed for their relative abundance at 48 and 72 h for all four treatment groups, as shown in Fig. 3. No significant differences (*p *> 0.05) were observed in the relative abundance of these transcripts between the four groups.

The relative expressions of all transcripts were compared between those treated and non-treated with IL-1β in the same group at the same time. We found almost all were different (*p *< 0.05), the relative expression of all genes being lower than in those non-treated with IL-1β.

## Discussion

Pro-inflammatory cytokines, such as IL-1β and other mediators produced by cytokine action on chondrocyte and synovial fibroblasts, may cause an imbalance in extracellular matrix (ECM) turnover, accelerate the degradation of the cartilage matrix, and also increase the incidence of chondrocyte apoptosis [18,19]. IL-1β appears to be first produced by the synovial membrane, and then diffuses into articular cartilage through the synovial fluid. It then activates chondrocytes, which in turn produce many catabolic factors. IL-1β has been implicated in the transcriptional upregulation of various MMPs, including *MMP-1 *[20,21] and *MMP-3 *[22,23]. For these reasons, this study used IL-1β as a typical inductor of an inflammatory metabolism. IL-1β was found to induce a distinct response in chondrosarcoma obtained from osteoarthritis chondrocytes. In this study, the viability and mitotic rate were shown to decrease, while the apoptosis rate increased significantly when treated with 10 ng/ml IL-1β. Moreover, proteoglycan production was significantly decreased by 20-30%. Gene expression of *MMP-3 *was upregulated 200%, *TIMP-3 *downregulated 50%, *HAS-1 *downregulated 60%, *HAS-2 *downregulated 90%, *AGG *downregulated 50%, and *COL2A1 *downregulated 90%. These results indicate that 10 ng/ml IL-1β can used to induce chondrosarcoma development in cartilage obtained from an OA joint. This system is suitable as a model for OA study in cell culture.

Numerous studies have demonstrated that MMPs are key enzymes involved in the destruction of articular cartilage in arthritic diseases [24]. The MMPs are an enzyme superfamily of at least 21 members, which can be classified into subgroups of collagenases (MMP-1, -8, -13), stromelysins (MMP-3, -10, -11), gelatinases (MMP-2, -9), and as membrane-type 1 (MMP-14) [25]. MMP-3 play the most important role in articular cartilage degradation [10]. They act to degrade the extracellular matrix (ECM): proteoglycans, gelatin, laminin, fibronectin and collagen (types III, IV and IX) [10]. In addition, MMP-3 can stimulate other enzymes in the MMP group, such as MMP-1, MMP-7, MMP-8, MMP-9 and MMP-13. This stimulation increases biochemical substance degradation including that of type II collagen, the most important type of collagen in ECM. The results of *MMP-3 *gene suppression found an 80% downregulation in *MMP-3 *gene expression in the *MMP-3 *siRNA group, significantly different from the control group (*p *< 0.05). This indicates that using siRNA interference could suppress *MMP-3 *gene expression. From a previous study, transfection of T/C-28a2 chondrocytes with double-stranded cathepsin B mRNA resulted in inhibition of cathepsin B biosynthesis by up to 70% due to RNA interference [26]. And NF-kBp65-specific siRNA can inhibit the expression of COX-2, NOS-2 and MMP-9 in IL-1β-induced and TNFα-induced chondrocytes [27]. These data suggest RNAi is an innovative method for sequence-specific, post-transcriptional gene silencing through cognate dsRNA. Thus RNAi targeting on *MMP-3 *may become an effective therapeutic method for osteoarthritis in the future.

It is well-known that the activity of MMPs is controlled by the tissue inhibitor of metalloproteinases (TIMP), a glycoprotein that inhibits all MMPs at a stoichiometry of 1:1 by forming high-affinity complexes [28]. An imbalance between MMPs and TIMPs is of great importance in the progression of OA [29,30]. Our study found that the *TIMP-3 *gene level in *MMP-3 *siRNA was no different from the control group. It is possible that silencing the *MMP-3 *gene has no effect on expression of *TIMP*.

*HAS-1 *and *HAS-2 *genes are capable of directing the synthesis of HA. HAS-*2 *was found to be the most abundant in articular chondrocytes, while synovial cells showed an opposite trend, with *HAS-1 *number levels always being more abundant than *HAS-2 *[31]. This study found that *MMP3*-siRNA has no effect on the expression of *HAS-1 *and *HAS-2*. Aggrecan was found to exhibit a unique feature in that the core protein had the capacity to interact with another GAG and HA [32]. This study found that silencing the *MMP-3 *gene had no effect on expression of the *AGG *gene. Collagens are a big family of proteins, the main one forming connective tissue in all higher animals. Connective tissue contains a mixture of cells, proteins, complex polysaccharides and inorganic constituents. The functional property of collagen type II is to give strength and flexibility to the connective tissue, resisting the tensions suffered in the direction of its fibers. Our study found that *MMP-3*-siRNA has no effect on expression of the *COL2A1 *gene.

The glycosaminoglycans consist of linear carbohydrate chains covalently linked to a protein core to form macromolecules termed proteoglycans. The substances most often classified as glycosaminoglycans include the following: hyaluronic acid (hyaluronan), chondroitin 4- and 6- sulfates, dermatan sulfate, keratin sulfate, heparan sulfate and heparin [33]. As mentioned above, the MMP-3 act to degrade proteoglycans. When the *MMP-3 *gene is suppressed, the degradation of proteoglycans could decrease. According to the results of this study, GAG and HA in the MMP-3 suppression group were significantly higher than in the other group.

Based on the cell apoptosis result, *MMP-3 *suppression could decrease chondrosarcoma apoptosis significantly. Previous experiments noticed that many hepatocellular carcinoma cells transfected with EGFP/aMMP-3 had fragmented nuclei characteristic of apoptosis. Data also suggest that the nuclear localization of MMP-3 is associated with an increased rate of apoptosis via its catalytic activity [34], one of study and inhibition of MMP activity rescues mammary epithelial cell apoptosis [35]. Furthermore, in some of modes of action the MMPs may alter the ECM microenvironment, leading to cell proliferation, apoptosis, or morphogenesis [36]. MMPs have also been shown to cause cell death. Proteinases or inappropriate ECM molecules induce apoptosis of mammary epithelial cells in culture, presumably through altered signaling from integrins [37]. Moreover, in the present study, the HA in the experimental group was increased. There was an experiment mentioned about the effect of HA that could protect chondrocyte apoptosis. HA protects against chondrocyte apoptosis during the development of OA, while it may not have definite effects on NO production in the joints. These inhibitory effects of HA on cell apoptosis may play a role in its mechanism of action in chondroprotection [38].

Our findings indicated that *MMP-3 *siRNA specifically decreased the expression of the *MMP-3 *gene, and led to decreased cell apoptosis, and increased cell viability and mitotis. Moreover, a suppression of *MMP-3 *can increase production of GAG and HA. For further study, *MMP-3 *gene suppression might be performed *in vivo*. If such gene suppression were successful *in vivo*, this method could play an important role in the treatment of osteoarthritis.

## Competing interests

The authors declare that they have no competing interests.

## Authors' contributions

KN carried out the study design, laboratory experiments (cell culture and gene expression) and coordination, and finished this manuscript. PK and PP carried out the biochemistry assay. SC, PSe and PC carried out the chondrosarcoma culture, morphology study and gene expression. All authors read and approved the final manuscript.
